# NIPBL Controls RNA Biogenesis to Prevent Activation of the Stress Kinase PKR

**DOI:** 10.1016/j.celrep.2015.12.012

**Published:** 2015-12-24

**Authors:** Kobe C. Yuen, Baoshan Xu, Ian D. Krantz, Jennifer L. Gerton

**Affiliations:** 1 Stowers Institute for Medical Research (SIMR), 1000 East 50^th^ Street, Kansas City, MO 64110, USA; 2 Children’s Hospital of Philadelphia, Division of Human Genetics, 3615 Civic Center Boulevard, Philadelphia, PA 19104, USA; 3 University of Kansas School of Medicine, Department of Biochemistry and Molecular Biology, 3901 Rainbow Boulevard, Kansas City, KS 66160, USA

## Abstract

NIPBL, a cohesin loader, has been implicated in transcriptional control and genome organization. Mutations in ***NIPBL***, cohesin, and its deacetylase ***HDAC8*** result in Cornelia de Lange syndrome. We report activation of the RNA-sensing kinase PKR in human lymphoblastoid cell lines carrying ***NIPBL*** or ***HDAC8*** mutations, but not ***SMC1A*** or ***SMC3*** mutations. PKR activation can be triggered by unmodified RNAs. Gene expression profiles in NIPBL-deficient lymphoblastoid cells and mouse embryonic stem cells reveal lower expression of genes involved in RNA processing and modification. ***NIPBL*** mutant lymphoblastoid cells show reduced proliferation and protein synthesis with increased apoptosis, all of which are partially reversed by a PKR inhibitor. Non-coding RNAs from an ***NIPBL*** mutant line had less m^6^A modification and activated PKR activity in vitro. This study provides insight into the molecular pathology of Cornelia de Lange syndrome by establishing a relationship between ***NIPBL*** and ***HDAC8*** mutations and PKR activation.

## Graphical Abstract

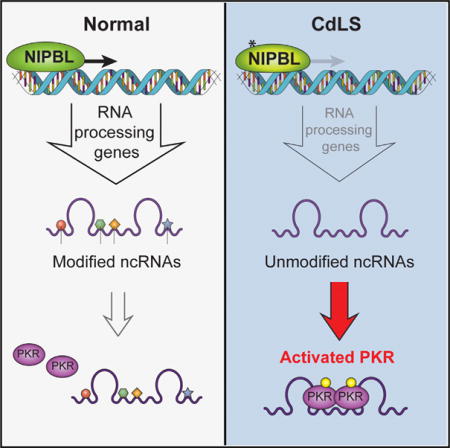

## INTRODUCTION

Chromosomes undergo structural changes to facilitate gene expression and genome organization. These changes are regulated, in part, by structural maintenance of chromosome (SMC) proteins. SMC proteins are evolutionarily conserved complexes that regulate the structural and functional organization of chromosomes from bacteria to humans (Nasmyth and Haering, 2005). SMC proteins are an essential component of complexes that organize chromosomes in the nucleus through the utilization of energy from ATP hydrolysis (Hirano, 2006). One of the SMC complexes, cohesin, is composed of four subunits including a heterodimer of SMC1A and SMC3 along with the kleisin RAD21. Cohesin generates cohesion of sister chromatids, which holds sister chromatids together from S phase until mitosis. The cohesin complex is crucial for various biological processes, such as chromosome segregation, condensation, gene expression, and double-strand break repair (Jeppsson et al., 2014).

The loading of cohesin complexes is facilitated by the loading factor Nipped B-like protein (NIPBL) or Scc2, a budding yeast ortholog. Genome-wide chromatin immunoprecipitation (ChIP) studies show that NIPBL co-localizes with both cohesin (Kagey et al., 2010) and condensin II (Dowen et al., 2013) complexes. Mutations in *NIPBL* lead to Cornelia de Lange syndrome (CdLS; OMIM: 122470; Krantz et al., 2004; Tonkin et al., 2004). CdLS is a genetic disorder distinguished by craniofacial dysmorphism, abnormal upper limb development, delayed growth, mild to severe cognitive impairment, and multiple organ malformations (Dorsett and Krantz, 2009). Together with CdLS, other multisystem developmental disorders resulting from mutations that affect cohesin, such as Roberts syndrome (RBS; OMIM: 268300), have been termed cohesinopathies. About 60% of CdLS cases are characterized by dominant heterozygous mutations in *NIPBL*. Mutations in *SMC1A*, *SMC3*, *HDAC8* (a cohesin deacetylase), and *RAD21* also cause CdLS or CdLS-like syndromes (Mannini et al., 2013). *NIPBL* mutations associated with CdLS are mostly loss-of-function mutations, and there is a positive correlation between the severity of the mutation and the phenotype (Mannini et al., 2013). Despite the importance of NIPBL in sister chromatid cohesion, cells derived from CdLS patients do not show high rates of aneuploidy (Kaur et al., 2005), indicating that the level of sister chromatid cohesion is sufficient for chromosome segregation. This raises the possibility that NIPBL may alter chromatin in a way that impinges on additional processes, and dysfunction in these processes underlies CdLS.

Emerging evidence indicates that cohesin and NIPBL have important functions in gene expression. In *Drosophila*, mutations in Nipped B affect the activation of homeobox genes that require long-distance interactions between enhancers and promoters, such as *cut* and *Ultrabithorax* (Rollins et al., 1999). Recently, it has been reported that NIPBL and Mediator regulate gene expression in developing limbs in zebrafish (Muto et al., 2014). A mutation in *SCC2* in budding yeast was associated with the loss of nucleosome-free regions (NFRs) at Scc2-bound genes (Lopez-Serra et al., 2014), providing a possible mechanism by which mutations in *SCC2* might affect multiple chromatin-based processes. The same mutation in *SCC2* was found to compromise the biogenesis of non-coding (nc)RNAs and translational fidelity (Zakari et al., 2015a). A previous study examining gene expression in lymphoblastoid cell lines (LCLs) derived from patients with CdLS suggested cohesin may promote gene expression (Liu et al., 2009). Results from these studies underscore the importance of NIPBL and cohesin as regulators of gene expression and further suggest CdLS may be caused by changes in gene expression (Zakari et al., 2015b). However, the precise molecular pathogenesis of CdLS is largely unclear.

We report here that the generation of aberrant RNAs may trigger the PKR-mediated stress response in LCLs derived from patients with CdLS. The activation of PKR is associated with reduced proliferation and protein synthesis and an increase in apoptosis. These defects are partially rescued by inhibiting PKR. Our results reveal that NIPBL supports a gene expression program that prevents the activation of the PKR kinase. Furthermore, PKR may be a useful target when considering possible therapies for CdLS.

## RESULTS

**Figure 1:**
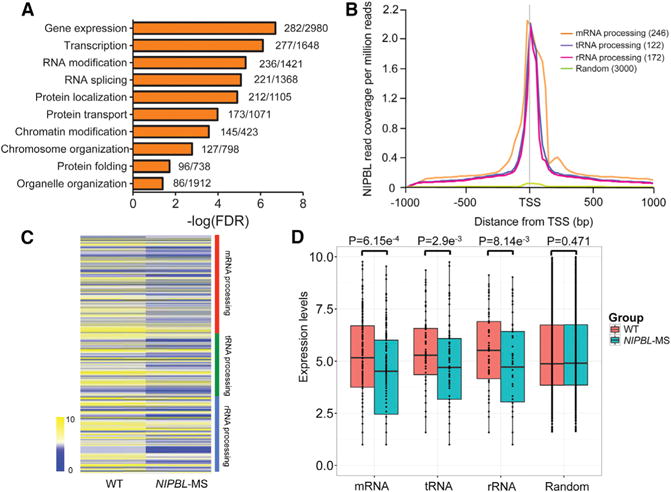
NIPBL Binds to and Regulates the Expression of RNA-Processing Genes in Human LCLs (A) Gene ontology (GO) analysis of the promoters/TSSs at which NIPBL binds in LCLs shows enrichment for genes involved in RNA modification and splicing. The × axis values (in logarithmic scale) correspond to the false discovery rate (FDR). The numbers next to each bar indicate the total number of genes differentially expressed of the total number of genes with that GO term. (B) NIPBL metagene-binding profiles at TSSs were generated using publicly available ChIP-seq data from LCLs for four gene groups (mRNA-, tRNA-, and rRNA-processing genes and random genes). Numbers in parentheses indicate the number of genes analyzed. (C) The heatmap shows the expression levels of mRNA-, tRNA-, and rRNA-processing genes in WT and *NIPBL*-MS LCLs. The average log_2_ expression value is displayed. (D) The data from (C) are shown as a bar plot along with the results of a t test. The expression of the group of random genes corresponding to those in (B) was not significantly different in WT and *NIPBL*-MS LCLs. See also Figures S1 and S2 and Table S1.

With over 60% of CdLS cases associated with *NIPBL* mutations, the etiology of CdLS can likely be at least partially elucidated by studying the loss of function of *NIPBL*. To investigate the potential functions of NIPBL, we first analyzed the publicly available data of ChIP followed by massive parallel deep sequencing (ChIP-seq) of NIPBL in human LCLs (Sequence Read Archive [SRA]: ERR139553). We examined the genes whose promoters are bound by NIPBL in LCLs with genome-wide gene ontology (GO) analysis. As shown in Figure 1A, the first few significantly enriched GO terms relate to gene expression and RNA modification. NIPBL firmly aligns with the transcription start site (TSS) of protein-coding genes in LCLs (Liu et al., 2009; Figure S1). Indeed, NIPBL has been implicated in gene expression (Dorsett and Merkenschlager, 2013). We decided to focus on RNA modification and processing.

**Figure 2:**
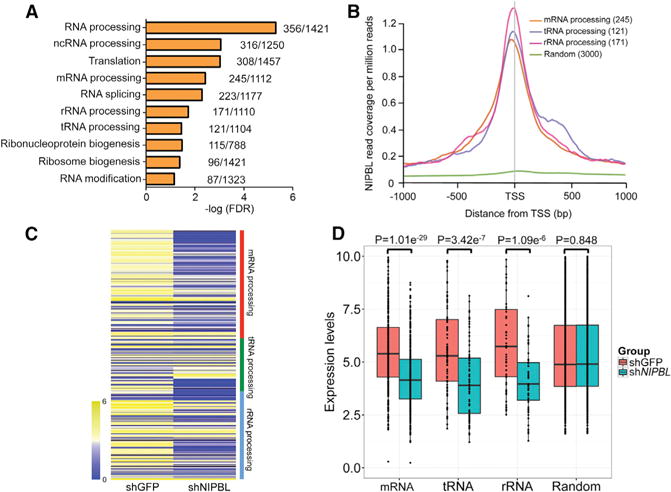
NIPBL Binds to and Regulates the Expression of RNA-Processing Genes in mESCs (A) GO analysis of the promoters/TSSs at which NIPBL binds shows enrichment for RNA-processing genes in mESCs. The × axis values (in logarithmic scale) correspond to the FDR. The numbers next to each bar indicate the total number of genes differentially expressed of the total number of genes with that GO term. (B) NIPBL metagene-binding profiles at TSSs were generated using publicly available ChIP-seq data from mESC for four gene groups (mRNA-, tRNA-, and rRNA-processing genes and random genes). NIPBL binds at the TSS of RNA-processing genes. Numbers in parentheses indicate the number of genes analyzed. (C) The heatmap shows the expression levels of mRNA-, tRNA-, and rRNA-processing genes after GFP (control) or *Nipbl* knockdown in mESCs. The average log_2_ expression value is displayed. (D) The data from the heatmap in (C) are shown as a bar plot along with the results of a t test. The expression of the group of random genes corresponding to those in (B) was not significantly different in GFP and *Nipbl* knockdown mESCs. See also Figure S3 and Table S2.

We previously reported that Scc2 is important for rRNA modification in budding yeast (Zakari et al., 2015a). We divided the RNA-processing genes into three different groups: mRNA-, tRNA-, and rRNA-processing genes. The binding of NIPBL to the promoter/TSS of various RNA-processing gene groups is depicted in the metagene analysis (Figure 1B). NIPBL preferentially binds to actively transcribed genes, with a positive correlation between its binding intensity at the promoter/TSS and expression levels of genes whose promoter/TSS are bound by NIPBL (Figure S1B). We also analyzed the publicly available NIPBL ChIP-seq data from mouse embryonic stem cells (mESCs). Consistently, the GO term analysis of genes whose promoter is bound by NIPBL in mESCs also indicates that NIPBL binds to the promoter of both the coding mRNA- and ncRNA-processing genes, such as tRNA-, mRNA-, and rRNA-processing genes (Figure 2A). In agreement with data from LCLs, NIPBL also binds to the promoter/TSS of genes involved in RNA processing in mESCs (Figure 2B). Together, these data suggest *NIPBL* binds at promoters of RNA-processing genes in mouse and human cells, giving it the potential to regulate expression of those genes.

We next asked whether NIPBL could promote expression of RNA-processing genes. We performed RNA sequencing (RNA-seq) of the LCL carrying an *NIPBL* missense (*NIPBL*-MS) mutation (6893G > A; R2298H) generated from an individual with CdLS together with a wild-type (WT) control line from a healthy individual. Overall, 2.7% of genes (880/32,994) were differentially expressed. As shown in Figure 1C, the expression of mRNA-, tRNA-, and rRNA-processing genes was reduced in the *NIPBL*-MS LCL. The reduced expression for each group was statistically significant (Figure 1D). Also, the GO term RNA processing or modification was significantly enriched for the downregulated genes (false discovery rate [FDR] < 3.18e–4). To confirm the lower expression of the RNA-processing genes in *NIPBL*-MS LCLs, we selected 12 RNA-processing genes from the heatmap in Figure 1C for qPCR and observed significantly decreased expression for all of them (Figure S2). We observed similar patterns of significantly reduced expression of the RNA-processing genes in mESCs with small hairpin RNA (shRNA) knockdown of *Nipbl* compared to that of GFP control (Figures 2C and 2D), by examining the publicly available data (Kagey et al., 2010). We wanted to confirm the reduced expression of RNA-processing genes in mESCs by qPCR. We performed shRNA-mediated knockdown of *Nipbl* in mESCs, and we confirmed the knockdown by both qPCR and western blotting (Figures S3A and S3B). We picked 12 RNA-processing genes from the heatmap in Figure 2C and confirmed their lower expression upon knockdown of *Nipbl* in mESCs (Figure S3C). NIPBL seems to be important for activating the expression of RNA-processing genes in LCLs and mESCs.

**Figure 3:**
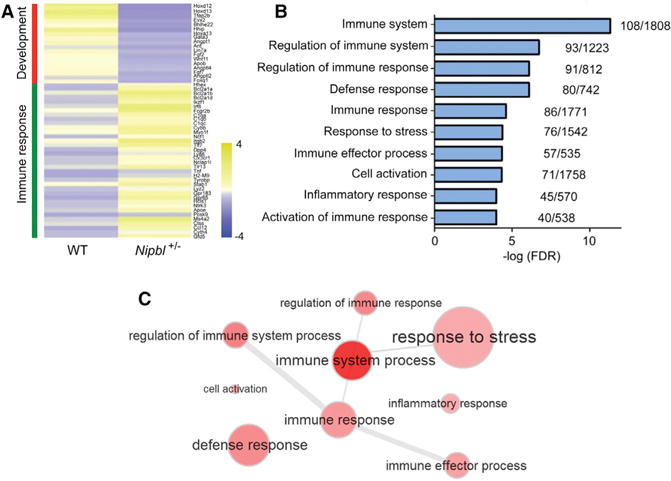
A Stress Response Signature in Nipbl^+/−^ MEFs (A) Heatmap showing the differential expression of genes between WT and mutant (n = 3). Key developmental genes and immune response genes are downregulated and upregulated, respectively, upon *Nipbl* haploinsufficiency. (B) Top 10 enriched GO terms associated with the 2-fold higher expressed genes in mutant MEFs are shown. (C) GO term clustering shows the inter-relationship between different GO terms for the higher expressed genes. The generality of the GO terms is indicated by the bubble radius, where larger bubbles represent broader terms and smaller bubbles imply more specific terms. The intensity of color represents the significance of enrichment, with darker indicating more significance. The thickness of the lines linking the GO terms reflects the significance of the relationship between them.

**Figure 4:**
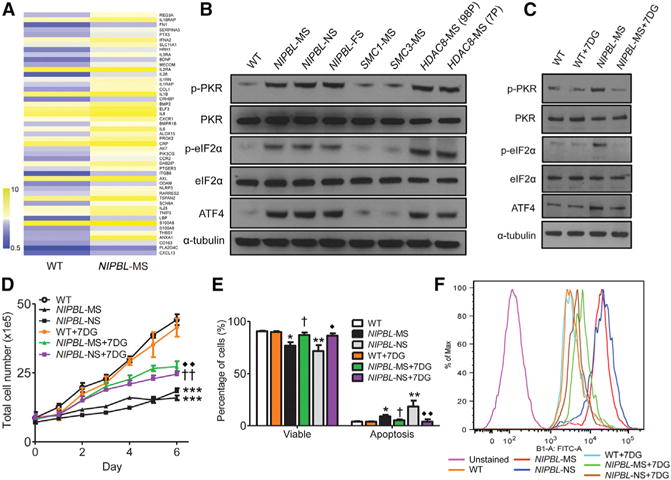
PKR and eIF2α Phosphorylation and Expression of ATF4 Are Elevated in NIPBL Mutant LCLs (A) The heatmap shows an upregulation of immune response genes in *NIPBL*-MS LCLs. The average log_2_ expression value is displayed. (B) There is increased p-PKR, p-eIF2α, and ATF4 in LCLs derived from patients with *NIPBL*-MS, -NS, and -FS mutations and *HDAC8* mutations (98P and 7P), but not from LCLs with cohesin *SMC1* missense (*SMC1*-MS) or *SMC3* missense (*SMC3*-MS) mutations. (C) 7DG treatment can inhibit the PKR-signaling cascade, as shown by reduced levels of phosphorylation of PKR and eIF2α as well as the reduced levels of ATF4. (D) The *NIPBL*-MS and *NIPBL*-NS LCLs show poor cell proliferation, which is partially rescued by treatment with 7DG (300 nM). Error bars represent SEM. (E) The *NIPBL*-MS and *NIPBL*-NS LCLs have elevated levels of apoptosis and lower viability, both of which are rescued by treatment with 7DG. (F) *NIPBL*-MS and *NIPBL*-NS LCLs have elevated levels of ROS, which are partially reversed by 7DG treatment. *p < 0.05, **p < 0.01, and ***p < 0.001 compared to WT; †p < 0.05 and ††p < 0.01 compared to *NIPBL*-MS; ◆p < 0.05 and ◆◆p < 0.01 compared to *NIPBL*-NS. All experiments were performed with n = 3–4. Error bars represent SEM. See also Figures S4 and S5.

A mouse model for CdLS that carries a heterozygous knockout of *Nipbl* has been developed previously (Kawauchi et al., 2009). To gain insights into the molecular etiology of CdLS, mouse embryonic fibroblasts (MEFs) were isolated from *Nipbl*^+/−^ mice and their WT littermates. We identified differential gene expression in the transcriptomes of WT and mutant MEFs by RNA-seq. Surprisingly, we observed a significant upregulation of immune stress response genes, manifested by some proinflammatory genes, toll-like receptors, and complement factors (Figure 3A). Consistently, GO term analysis indicated that terms associated with immune stress response were the most significantly enriched (Figure 3B). The inter-relationship between the enriched GO terms is depicted in the clusters shown in Figure 3C, with the generality of GO terms being immune response and stress response. Taken together, the results indicated that *Nipbl*^+/−^ MEFs exhibit an upregulation of immune stress response. Consistent with the *Nipbl*^+/−^ MEFs, we observed a significant upregulation of immune response genes in the *NIPBL*-MS LCL (FDR < 5.43e–3) (Figure 4A). Surprised by the results, we wondered if this signature was connected to the generation of aberrant RNAs.

We speculated that the upregulated immune signature could be a causal effect of the downregulation of RNA-processing genes. It has been reported that RNA with less processing or modification could lead to an immune response. In fact, modifications in RNA provide a basis for various immune sensors to distinguish between self-RNAs and pathogenic RNAs (Nallagatla et al., 2008). We hypothesized that the decreased expression of RNA-processing genes could generate a stress response due to the generation of RNAs with processing, modification, or folding defects, thereby causing an immune response. A central player in sensing aberrant RNAs is the interferon-induced protein kinase PKR, which is also a key component for innate immunity (Nallagatla et al., 2007). Indeed, we found that the activity of PKR, indicated by phosphorylated PKR (p-PKR), was increased in *NIPBL*-MS cells and also in other LCLs carrying an *NIPBL* frameshift mutation (*NIPBL*-FS) or an *NIPBL* nonsense mutation (*NIPBL*-NS) (Figure 4B).

PKR is one of four mammalian kinases that phosphorylate eukaryotic initiation factor 2-α subunit (eIF2α) in response to stress signals. PKR is activated mainly in response to viral infection (Holcik and Sonenberg, 2005). PKR is a key component of innate immunity that recognizes and binds to pathogenic RNAs. The interaction of RNAs with PKR promotes and stabilizes its dimerization. PKR then undergoes auto-phosphorylation and subsequently phosphorylates eIF2α to shut off general translation, while translation of the ATF4 stress response transcription factor is upregulated (Hinnebusch, 2005). Consistently, the levels of peIF2α and ATF4 in *NIPBL* mutant LCLs were upregulated (Figure 4B), further suggesting that the PKR-signaling cascade was activated in the mutant LCLs. We found that the PKR-signaling cascade also was elevated in *Nipbl*^+/−^ MEFs (Figure S4). Additionally, a similar signature for p-PKR, p-eIF2A, and ATF4 was observed in LCLs carrying *HDAC8* missense (7P and 98P) mutations (958G > A; G320R and 539A > G; H180R, respectively) (Figure 4B). Interestingly, the PKR signature was not observed in LCLs carrying a mutation in *SMC1A* (1487G > A; R496H) or *SMC3* (1464–1466del) (Figure 4B). These results suggest PKR activation may be associated with some CdLS mutations, but not others. These results may provide a molecular distinction between SMC mutations and mutations in the SMC regulators *HDAC8* and *NIPBL*.

Increased PKR activity inhibits translation by blocking the initiation of protein synthesis through the phosphorylation of eIF2α. Thus, pharmacological inhibition of PKR could represent an attractive strategy for restoring translation. Inhibitors of PKR have been identified, including 7-desacetoxy-6,7-dehydrogedunin (7DG). The specificity of 7DG has been tested and confirmed; mouse cells treated with 7DG phenocopy cells with RNAi knockdown of PKR (Hett et al., 2013). Our western blot results indicated that 7DG can inhibit the PKR-signaling pathway by reducing the levels of p-PKR and eIF2α and the expression of ATF4 in the *NIPBL*-MS LCL (Figure 4C). Moreover, ^35^S methionine metabolic labeling assays showed a significant increase in protein synthesis in the *NIPBL*-MS and *HDAC8* mutant LCLs upon treatment with 7DG (Figure S5A).

We treated LCLs with 7DG to test for additional rescue effects. It has been shown that the activation of PKR induces apoptosis (Srivastava et al., 1998). As shown in Figures 4D and 4E, both the *NIPBL*-MS and the *NIPBL*-NS LCLs demonstrated reduced growth in culture, with a concomitant propensity to undergo apoptosis. The *NIPBL*-FS had no obvious growth or proliferation phenotype (data not shown). We treated *NIPBL*-MS and *NIPBL*-NS LCLs with 7DG. As shown in Figures 4D and 4E, both the slower proliferation and the elevated levels of apoptosis in the *NIPBL*-MS and *NIPBL*-NS LCLs were significantly reversed by 7DG (300 mM). The partial, but remarkable, rescue of *NIPBL*-MS and *NIPBL*-NS LCLs by 7DG indicated that PKR activation contributes significantly to the phenotypes associated with the *NIPBL* mutant LCLs. Moreover, 7DG could significantly attenuate the increased levels of reactive oxygen species (ROS) present in the *NIPBL*-MS and *NIPBL*-NS LCLs (Figure 4F). These results raise an exciting possibility that PKR inhibition may have potential therapeutic value in the management of CdLS.

To test the hypothesis that aberrant RNAs are a source of stress in the *NIPBL* mutant LCLs, we sought to further characterize RNA biogenesis. Since PKR directly interacts with RNA (Hinnebusch, 2005), we hypothesized that there would be increased levels of aberrant RNAs in *NIPBL* mutant LCLs caused by defects in RNA processing, thus activating PKR. Since RNAs undergo extensive chemical modifications (Cantara et al., 2011), we reasoned that RNAs from *NIPBL* mutant LCLs may have modification defects. Unmodified RNAs are potent activators of PKR (Nallagatla and Bevilacqua, 2008). We examined one modification in particular, *N*^6^-methyladenosine (m^6^A), an adenosine modification catalyzed by methyltransferases such as METTL3 and METTL14 (Liu et al., 2014). m^6^A was selected for study because it exists in most of the RNAs in a variety of organisms. The abundance of m^6^A throughout the transcriptome (about 7,000 mRNAs and over 300 ncRNAs in the mammalian genome) has been demonstrated by m^6^A profiling from two independent studies (Dominissini et al., 2012; Meyer et al., 2012). Knockdown of either *METTL3* or *METTL14* results in a reduction in total m^6^A levels in human cells (Dominissini et al., 2012; Liu et al., 2014).

**Figure 5:**
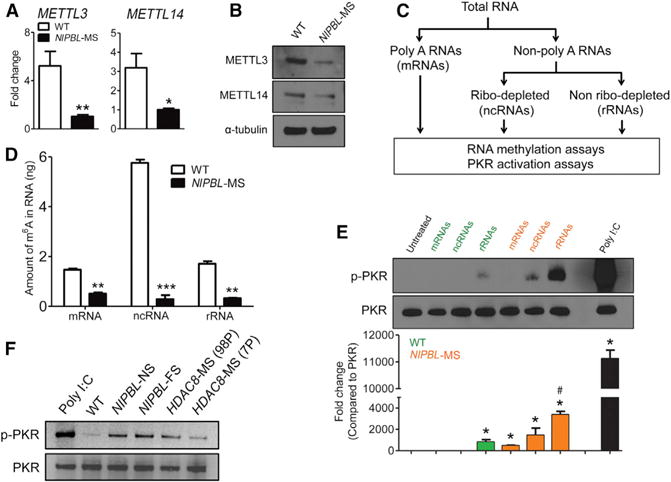
RNA Derived from NIPBL-MS Cells Contains Less m^6^A Modification and These RNAs Activate PKR In Vitro (A) qPCR shows the lower expression of *METTL3* and *METTL14* RNA in *NIPBL*-MS cells. Ubiquitin C served as a loading control. *p < 0.05 and **p < 0.01 compared to WT. (B) The reduction of METTL3 and METTL14 protein expression is shown in western blots. (C) The scheme used to fractionate RNA is diagrammed. The mRNAs are first isolated from the total RNA, followed by ncRNAs and rRNAs (see Experimental Procedures for details). (D) m^6^A levels are significantly reduced in mRNAs, ncRNAs, and rRNAs from *NIPBL*-MS cells compared to WT cells. There is an especially dramatic decrease (more than 6-fold) in m^6^A levels in the ncRNAs of the *NIPBL* mutant LCLs. **p < 0.01 and ***p < 0.001 compared to WT. (E) Both ncRNAs and rRNAs isolated from *NIPBL*-MS LCLs are capable of activating recombinant PKR in vitro; 10 ng Poly I:C was used as a positive control for PKR activation. *p < 0.001 compared to untreated control; #p < 0.001 compared to WT rRNA. (F) Total RNA isolated from *NIPBL*-NS and *NIPBL*-FS, and *HDAC8* (98P) and *HDAC8* (7P) can induce PKR activation in vitro; 1 ng Poly I:C was used as a positive control for PKR activation. For (E) and (F), 10 ng RNA was used in each reaction.

We found that the expression of both *METTL3* and *METTL14* was downregulated in the *NIPBL*-MS LCL compared to a WT LCL (Figures 5A and 5B). There was no significant difference in METTL3 and METTL14 protein levels among *SMC1A*-MS, *SMC3*-MS, and WT LCLs (Figure S5B), which is consistent with the absence of PKR activation in *SMC1A*-MS and *SMC3*-MS LCLs. Given the reduced expression of both m^6^A methyltransferases in the *NIPBL*-MS LCL, we then tested whether RNAs derived from this line had lower levels of m^6^A modification. We first performed RNA fractionation outlined in Figure 5C to separate the total RNA into three main groups as follows: (1) mRNAs containing a polyA tail; (2) rRNAs (>80%) and some ncRNAs following removal of mRNA; and (3) ncRNAs, for example, tRNAs, small nuclear RNAs (snRNAs), and small nucleolar RNAs (snoRNAs), after removal of mRNA and rRNA through ribo-depletion. We then measured m^6^A levels in the three groups of RNAs using an ELISA-based methylation detection method. We found that all three groups of RNAs isolated from the *NIPBL*-MS LCL showed significant reduction in m^6^A modification compared to the same groups of RNAs isolated from a WT LCL (Figure 5D). The decrease was especially striking (more than 6-fold) in the ncRNA fraction, which would include tRNAs. tRNAs are the most highly modified RNA species (Phizicky and Hopper, 2010) and would, therefore, be most likely to show a defect.

We further studied whether the three different RNA fractions isolated from the *NIPBL*-MS LCLs stimulated PKR in vitro. We performed PKR activation assays with 10 ng RNA from each fraction, using poly I:C as a positive control for PKR activation (Figure 5E). We found that the rRNA fraction from the *NIPBL*-MS LCLs was a strong activator of PKR, followed by the ncRNAs. The p-PKR/PKR ratio of *NIPBL*-MS rRNA (lane 7) compared to WT rRNA (lane 4) was 5.6 ± 1.3 higher (p < 0.001). The results suggested that rRNAs from the *NIPBL*-MS LCL are potent activators of PKR. The activation could be based on the lack of m^6^A modification, other modifications, or even misfolding (Heinicke and Bevilacqua, 2012), due to the underexpression of various genes involved in RNA modification and processing.

We next tested whether RNA from other *NIPBL* or *HDAC8* mutant LCLs could activate PKR activity in vitro. Since rRNAs from the *NIPBL*-MS LCL most significantly activate PKR and rRNAs represent the majority of RNA in cells, total RNA was used in these in vitro assays. As shown in Figure 5F, total RNA from *NIPBL*-NS and *NIPBL*-FS, as well as from both *HDAC8* mutant cell lines, could activate PKR. Taken together, defects in RNA biogenesis may, therefore, serve as an underlying mechanism to activate the PKR-mediated stress response in *NIPBL* and *HDAC8* mutant LCLs.

## DISCUSSION

**Figure 6:**
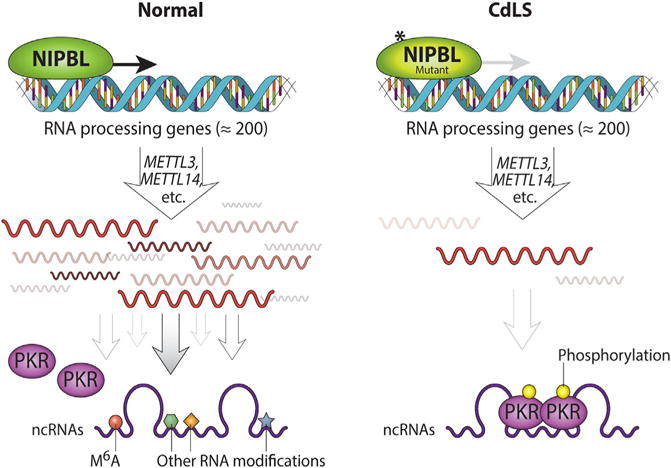
Model for the Activation of PKR in LCLs Derived from Individuals with Mutations in *NIPBL* NIPBL binds to the promoter/TSS of RNA-processing genes, including *METTL3* and *METTL14*, to promote their expression. The RNA-processing genes are essential for the RNA modifications such as m^6^A methylation (red circle), pseudouridylation, etc. In normal cells, RNAs are highly modified with m^6^A methylation and other modifications to prevent activation of PKR. However, in CdLS LCLs with loss of NIPBL function, RNA-processing genes are expressed at lower levels. RNAs are generated that contain less m^6^A modification and potentially other modifications as well. Such aberrant RNAs cause the activation of PKR that is marked by both dimerization and auto-phosphorylation.

The data presented here are consistent with a working model in which NIPBL activates the expression of RNA-processing genes to promote RNA biogenesis (Figure 6). With the loss of *NIPBL* function, either via mutation or knockdown, these genes are expressed at lower levels. Defects in RNA biogenesis, including lower levels of m^6^A modification, lead to the activation of PKR, triggering a stress response. Inhibition of PKR with 7DG provides partial repression of that stress response. We have therefore identified a previously unknown pathway that could contribute to the molecular etiology of CdLS. Related cellular stress pathways have been shown to contribute to pathogenesis in Alzheimer’s and Huntington’s diseases. The results of our study (1) provide insights into the role of *NIPBL* in ncRNA biogenesis and (2) reveal that RNA biogenesis defects, such as lower levels of m^6^A methylation, could trigger stress associated with *NIPBL* mutations.

This study suggests that stress created by defects in RNA biogenesis and an upregulation of PKR activity may be a contributing factor for cellular defects in LCLs derived from patients with CdLS. Eukaryotic RNAs are demarcated with a variety of modifications, including 5mC, m^5^U, s^2^U, m^6^A, ψ, or 2′-O-methylation, which mark them as self, as compared to the unmodified RNAs from viruses and bacteria, which can be identified as non-self. For example, human rRNA has ten times more pseudouridine (ψ) and 25 times more 2′-O-methylated nucleosides than bacterial rRNA (Margulis and Chapman, 1998). This striking difference in modifications helps account for why bacterial and viral RNAs are immunogenic, even though they have the same chemical structure as human RNAs. Previous studies in vitro in human dendrite cells (Karikó et al., 2005) and in vivo in mice (Kormann et al., 2011) confirmed that RNA with modifications could significantly suppress immune responses and inflammatory cytokine formation compared to unmodified RNA. In addition to the downregulation of the genes encoding the methylation enzymes METTL3 and METTL14, in both the *NIPBL*-MS LCL and the shRNA knockdown of *Nipbl* in mESCs, genes encoding the enzymes needed for pseudouridylation were significantly downregulated, suggesting that the absence of multiple modifications or defects may synergize in the activation of PKR. It will be interesting to explore and characterize additional RNA biogenesis defects and how they contribute to cellular phenotypes in CdLS in the future.

RNA modifications such as m^6^A serve various functions in regulating cellular processes. For example, it has been proposed that m^6^A methylation maintains stem cell pluripotency by promoting the decay of RNAs encoding developmental regulators (Wang et al., 2014). Knockdown of *Mettl3* and *Mettl14* in mESCs results in the loss of their self-renewal ability (Wang et al., 2014). Similar phenotypes, upon *Nipbl* knockdown, have been reported, including differentiation and increased expression of differentiation genes (Kagey et al., 2010). These results suggest that the cell differentiation induced by *Nipbl* knockdown may be partially due to the lack of m^6^A methylation.

NIPBL and cohesin may contribute to gene expression in different ways. For instance, NIPBL may be involved in the maintenance of NFRs, while cohesin may be important in long-distance interactions. Due to these different molecular functions, loss of function may not have equivalent effects on gene expression. For example, the gene expression profiles of cells upon *NIPBL* or cohesin knockdown are different (Muto et al., 2011; Zuin et al., 2014). Our study further supports this idea since CdLS LCLs with mutations in *SMC1A* or *SMC3* do not show PKR activation. A previous study showed that NIPBL directly interacts with histone-deacetylating enzymes HDAC1 and HDAC3 in human cells (Jahnke et al., 2008), suggesting that NIPBL may initiate the chromatin-remodeling processes through the recruitment of these HDACs in transcriptional regulation. The budding yeast ortholog of *NIPBL*, *SCC2*, may participate in transcriptional regulation by maintaining NFRs through the association with remodels the structure of chromatin (RSC; Lopez-Serra et al., 2014). In the future, it will be important to continue to dissect the molecular role of NIPBL and cohesin in gene expression, since this knowledge will help us understand how loss of function leads to human disease.

In summary, we suggest that NIPBL facilitates a gene expression program compatible with normal RNA biogenesis. Upon *NIPBL* loss of function, there is reduced expression of RNA-processing genes, which correlates with the generation of unmodified RNAs, including m^6^A deficiency. Such aberrant ncRNAs could activate the PKR-signaling cascade, leading to poor cell proliferation, protein synthesis, and apoptosis. Importantly, treatment with a PKR inhibitor can partially rescue these defects. The findings shed light on the molecular etiology of CdLS by highlighting the activation of PKR in the *NIPBL* and *HDAC8* mutant cells. Identification of elevated PKR activity suggests a new avenue for disease management, namely the use of PKR inhibitors to ameliorate cellular stress associated with CdLS.

## EXPERIMENTAL PROCEDURES

### qRT-PCR and RNA-Seq Analysis

Total RNA from LCLs and mESCs was isolated with TRIzol Reagent (Life Technologies, 15596) following the manufacturer’s instructions. RNA was treated with DNase I (New England Biolabs, M0303S) to remove contaminating genomic DNA. cDNA was synthesized with iScript cDNA Synthesis Kit (Bio-Rad, 170-8890). The cDNA was then mixed with primers and Power SYBR Green Master Mix (Life Technologies, 4367659). The gene expression levels were determined by the Applied Biosystems 7900HT Fast Real-Time PCR System (Life Technologies), followed by normalization to the housekeeping genes ATP synthase β-subunit (ATP5B) and ubiquitin C (UBC). See Tables S1 and S2 for primers used for human and mouse, respectively.

For RNA-seq of LCLs and MEFs, total RNA was depleted of rRNA with the Ribo-Zero kit (Epicenter). The ribo-depleted RNA samples were amplified with the TruSeq RNA Sample Prep Kit (Illumina) for Solexa sequencing. Reads from two biological replicates for WT and *NIPBL*-MS were aligned to the human genome UCSC hg19 and to gene annotation from Ensembl 78 using TopHat 2.0.10 (Trapnell et al., 2009). Similarly, reads from three biological replicates for WT and *Nipbl*^+/−^ were aligned to the mouse genome UCSC mm10 and to gene annotations from Ensembl 72 using TopHat 2.0.10. For both LCLs and MEFs, the differential expression analysis at FDR < 0.05 and the assessment of statistically significant read coverage for each gene were performed with edgeR (Robinson et al., 2010).

### ChIP-Seq Analysis

For LCLs, reads from ChIP-seq experiments were aligned to the human genome UCSC hg19 using Bowtie2 aligner 2.1.0, allowing uniquely mapped reads only up to two mismatches (Langmead et al., 2009). For mESCs, reads from ChIP-seq experiments were aligned to the mouse genome UCSC mm10 using Bowtie2 aligner 2.1.0, allowing uniquely mapped reads only up to two mismatches (Langmead et al., 2009). For both LCLs and mESCs, reads were extended to 150 bp toward the interior of the sequenced fragment and normalized to total reads aligned. The average coverage was binned in 25-bp intervals. Peak calling was performed using MACS 2.0.10 (Zhang et al., 2008) with stringent conditions to determine statistical enrichment at an FDR < 1e–9, resulting in high-confidence peaks that were used for subsequent analysis and for depicting enrichment profiles. NIPBL peaks spanning 2 kb on both sides of the TSSs were binned into 100-bp windows for analysis. Peak annotation was done using HOMER algorithm (Heinz et al., 2010). GO analysis was performed using DAVID (Huang da et al., 2009). The background GO terms were the union of biological processes, cellular components, and molecular functions. An FDR cutoff of 0.01 was used to select enriched terms. GO term clustering was done using REVIGO (Supek et al., 2011).

### Generation of LCLs

Human LCLs were generated from patients with mutations in *NIPBL*, *SMC1A*, *SMC3*, or *HDAC8* under an IRB-approved protocol of informed consent. The mutations were identified by sequencing (Liu et al., 2009).

### Cell Culture Conditions

#### Human LCLs

LCLs were grown in a T25 flask with RPMI media supplemented with 20% fetal bovine serum (FBS). Fresh media were added daily for expansion.

#### mESCs

V6.5 mESCs (Novus Biologicals) were grown on irradiated MEFs. Cells (8.6 × 10^6^) were grown on 0.1% gelatinized (STEMCELL Technologies, 07903) 150-mm tissue culture plates in ESC-c medium consisting of the following: DMEM supplemented with 15% FBS (HyClone, SH30070.03); 1× b-mercaptoethanol (Millipore, ES-007-E); nonessential amino acids (STEMCELL Technologies, 07600); 1× GlutaMAX (STEMCELL Technologies, 07100); and 50 μg/ml penicillin/streptomycin (STEMCELL Technologies, 07500).

#### Irradiated MEFs

Low-passage irradiated MEFs were grown on 150-mm tissue culture plates 48 hr prior to seeding the mESCs in the ESC-c media described above.

### MEFs Isolation and Culture

Embryos (14.5 days post-coitum [DPC]) were dissected from one pregnant mouse and the embryo’s limbs, brain, and internal organs were carefully removed. The rest of the embryos were then minced into small pieces with a sterile surgical blade. The minced embryo was then incubated in a 50-ml tube with 3 ml trypsin for ~30 min at 37°C with the occasional stir. MEF media (10 ml) were added to the 50-ml tube and mixed well before plating onto a gelatinized 10-cm tissue culture dish. After 3–5 days of culture, all cells were frozen down at 2 × 10^6^ per vial at post-natal day (P)1. MEFs were grown in DMEM (Sigma, D6546) supplemented with 10% FBS (Gibco, 10437-077). Medium was changed every 2 days. Cells from the third passage were used for RNA isolation for sequencing.

### Lentivirus-Based RNAi Knockdown in mESCs

Lentiviral particle preparation and infection were performed as previously described with some modifications (Lin et al., 2013). Briefly, 70% confluent HEK293T cells in a 150-mm tissue culture plate were co-transfected with 8 μg mouse *Nipbl* shRNA construct (Open Biosystems, TRCN0000124037) or GFP shRNA (Addgene, 30323), 6 μg psPAX2 packaging plasmids (Addgene, 12260), and 2 μg pMD2.G envelop plasmids (Addgene, 12260) with 40 μl Lipofectamine 2000 (Life Technologies, 11668027). The ESC-c medium was replaced after 16 hr of transfection. The medium containing lentiviral particles was collected 48 and 72 hr after the transfection. The medium was filtered through 0.45-μm syringe filters (Nalgene) and concentrated by ultra-centrifuge at 25,000 rpm in an SW-41Ti rotor (Beckman Coulter) for 2 hr at 4°C. The V6.5 mESCs were infected with concentrated lentiviral particles in ESC-c medium containing 8 μg/ml polybrene (Sigma, H9268). Then 24 hr after infection, the media were replaced with 2 μg/ml puromycin (InvivoGen, ant-pr-1) for 5 days to select for stable integration of the shRNA construct. The medium with puromycin was changed daily. The GFP and NIPBL knockdown cells were grown one passage off feeders before harvesting for protein extraction and RNA isolation.

### Western Blots

Western blots were performed as described previously (Yuen et al., 2011). Briefly, the whole-cell extracts from mESCs or LCLs were isolated by ice-cold lysis buffer consisting of the following: 50 mM HEPES (pH 7.9), 5 mM MgCl_2_, 0.2% Triton, 20% Glycerol, 300 mM NaCl, and proteinase inhibitor cocktail tablet (Roche, 04693116001). The lysates were incubated on ice for 30 min and then centrifuged at 20,000 × *g* for 20 min at 4°C. The supernatant was collected and analyzed for protein concentration using the Lowry method (Bio-Rad, 500-0111). For each sample, 25 μg total protein was electrophoresed under reducing conditions through a NuPAGE 4%–12% Bis-Tris protein gel (Life Technologies, NP0322BOX). The resolved proteins were electroblotted on an immobilon-P polyvinylidene difluoride membrane (Millipore, IPVH00010) using wet transfer at 100 V for 90 min at 4°C. The membranes were blocked with 1% BSA in 0.5% Tween-20 PBS (PBST) for 60 min before an overnight incubation with primary antibodies at 4°C. The membranes were then probed with a horseradish peroxidase-conjugated secondary antibody at a dilution of 1:3,000 for 1 hr at room temperature. The membranes were developed with an enhanced chemiluminescence detection system (ECL reagents; Thermo Scientific, 32132) and then exposed to X-ray films. The signal intensities were quantified using ImageJ (NIH) and normalized with the housekeeping protein α-tubulin.

### Apoptosis Assays

Annexin V was used to study apoptosis of LCLs. First, WT and *NIPBL*-MS and *NIPBL*-NS cells were washed in cold PBS and pelleted by centrifugation, followed by re-suspension with annexin-binding buffer consisting of the following: 10 mM HEPES, 140 mM NaCl, and 2.5 mM CaCl_2_. The cells were then stained with DAPI for 10 min at room temperature. After that, 5 μl annexin V conjugated with Alexa Fluor 488 dye (Life Technologies, A13201) was added to the cell suspension and incubated at room temperature for 15 min. The stained cells were assayed quickly with MACSQuant (Miltenyi Biotec). Data analysis was performed with FlowJo software (Tree Star).

### ROS Assays

The levels of ROS in WT, *NIPBL*-MS, and *NIPBL*-NS cells, treated with 300 nM 7DG or untreated for 24 hr were determined with the DCFDA-Cellular Reactive Oxygen Species Detection Assay Kit (Abcam, ab113851), following the manufacturer’s instructions. Briefly, cells were washed in PBS, followed by staining with 20 μM DCFDA and incubation for 30 min at 37°C. The stained cells were analyzed immediately using MACSQuant at excitation 485 nm/emission 535 nm. Data analysis was performed with FlowJo.

### RNA Fractionation

Total RNAs from WT and *NIPBL*-MS LCLs were isolated with TRIzol Reagent. First the RNAs with polyA tails were separated from the rest of the RNA using the polyA spin mRNA isolation kit (NEB, S1560S), following the suggested protocol. The resulting RNA that mainly consisted of rRNA was divided into two portions. One portion of this RNA sample was subjected to ribo-depletion using the Ribo-Zero rRNA Removal kit (Epicenter, MRZH116), resulting in a pool of RNAs enriched for ncRNAs such as tRNAs, microRNAs, and snoRNAs.

### m^6^A Methylation Assays

To perform m^6^A methylation assays, 200 ng of each mRNA, ncRNA, and rRNA fraction was used. The m^6^A methylation levels of the RNA fractions from WT and *NIPBL*-MS cells were determined using the EpiQuik m6A RNA Methylation Quantification Kit (Epigentek, P-9005-48), according to the manufacturer’s instructions. Briefly, a standard curve was prepared by making six different concentrations of the positive control, ranging from 0.01 to 0.5 ng/μl. RNA samples were added to the strip wells anchored on a 96-well plate. The plate was gently tilted and shaken several times to allow the RNA to bind evenly to the bottom of the wells. The plate was then sealed and incubated at 37°C for 90 min. After that, the wells were washed three times with 150 μl washing buffer. Capture antibody diluted 1:1,000 was added to the wells and incubated at room temperature for 60 min, followed by washing three times with 150 μl washing buffer. Detection antibody with 1:2,000 dilution was then added to each well for detecting the antibody. The plate was incubated at room temperature for 30 min, followed by washing four times with 150 μl washing buffer. Enhancer solution diluted 1:5,000 was added to each well and incubated at room temperature for 30 min, followed by washing five times with 150 μl washing buffer. Detection solution (100 μl) was then added to each well and incubated at room temperature away from light for 10 min. After that, 100 μl stop solution was added to quench the enzyme reaction. The absorbance was taken with a microplate reader at 450 nm within 15 min. The amount of m^6^A was calculated with the following equation:

$$m^{6}A\left(\text{ng}\right)=\frac{\text{OD}:\text{Sample}-\text{OD}:\text{Background}}{\text{slope of standard curve}}$$

### PKR Activation Assays In Vitro

PKR activation assays were performed as described (Zheng and Bevilacqua, 2004). RNAs from WT and mutant LCLs were tested for the ability to directly activate PKR protein in vitro, which was determined by levels of PKR phosphorylation in western blots. Briefly, 0.1 ng recombinant PKR (Life Technologies, PV4821) was dephosphorylated by treating with λ-PPase (NEB, P0753S) for 30 min at 37°C. λ-PPase was inactivated by treatment with freshly prepared sodium orthovanadate. The dephosphorylated PKR was then incubated with 10 ng RNA from WT and *NIPBL*-MS, *NIPBL*-NS, *NIPBL*-FS, and *HDAC8* mutant cells in the activation buffer (20 mM HEPES [pH 7.5], 4 mM MgCl_2_, 100 mM KCl, and 1 mM ATP) for 3 hr at 30°C. Reactions were stopped by adding SDS loading buffer and PKR was resolved on a NuPAGE 4%–12% Bis-Tris protein gel. The phosphorylation of PKR was determined with p-PKR antibodies (Abcam, ab32036).

### Cell Proliferation Assays

WT, *NIPBL*-MS, and *NIPBL*-NS LCLs (3 × 10^5^) were set in a six-well plate with RPMI with 20% FBS; 300 mM 7DG was supplied in the medium for some LCLs; and 10 μl cells was used to perform cell counting using the TC20 Automated Cell Counter (Bio-Rad, 145-0102) daily for 6 days. The experiments were done in triplicate.

### ^35^S methionine Metabolic Labeling Assays

The metabolic labeling assays for proteins have been described previously (Xu et al., 2013). Briefly, WT and *NIPBL*-MS LCLs were washed in PBS twice; switched to 3 ml Met/Cys-free RPMI containing 10 μM MG-132, a proteasome inhibitor; and pulsed with 30 μCi ^35^S-methionine. Cells were lysed in RIPA buffer (50 mM Tris [pH 7.2], 150 mM NaCl, 1% sodium deoxycholate, 0.1% SDS, 1% Triton X-100, 10 mM NaF, and 1 mM Na3VO4). Proteins were precipitated by the addition of hot 10% trichloroacetic acid. After centrifugation, the precipitate was washed twice in acetone. The precipitate was dissolved in 100 μl 1% SDS and heated at 95°C for 10 min. An aliquot of the SDS extract was counted in Ecoscint for ^35^S radioactivity in a liquid scintillation spectrometer to determine the amount of ^35^S-methionine incorporated into proteins.

### Antibodies

Primary antibodies for NIPBL were purchased from Bethyl (A301-799A); p-PKR (ab32036), METTL3 (ab49253), METTL14 (ab98166), and α-tubulin (ab15246) were purchased from Abcam; PKR was purchased from Santa Cruz Biotechnology (sc-6268); and p-eIF2α (3398), eIF2α (9722), and ATF4 (11815) were purchased from Cell Signaling Technology.

### ChIP-Seq Data Analyzed in This Study


ChIP-Seq DatasetAccession NumberReference
Human LCL NIPBLSRA: ERR139553Mapping of the cohesin loading factor NIPBL in the human genome yields insights in Cornelia de Lange syndrome (I.D.K., unpublished data)
mESC NIPBLGEO: GSE22562Kagey et al. (2010)


### Statistical Analysis

All experiments were repeated independently at least in triplicate, and the data are presented as mean ± SD. Statistical significance was determined using the Student’s t test. A p value of < 0.05 was considered to be statistically significant.

## Supplementary Material

Yuen et al Supplm File

## Highlights

- NIPBL-deficient LCLs and mESCs have lower expression of RNA-processing genes
- NIPBL and HDAC8 mutant LCLs have activated PKR
- RNAs derived from NIPBL and HDAC8 mutant LCLs activate PKR in vitro
- PKR activation may contribute to the molecular pathology of CdLS
