# Supplementary material for: NIPBL Controls RNA Biogenesis to Prevent Activation of the Stress Kinase PKR
Source: Cell Rep. Author manuscript; Available in PMC 2016 Jun 13. (PMC4904785; doi:10.1016/j.celrep.2015.12.012)
Supplement: Yuen et al Supplm File [file NIHMS790245-supplement-Yuen_et_al_Supplm_File.pdf]

Cell Reports

Supplemental Information

NIPBL Controls RNA Biogenesis to Prevent Activation of the Stress Kinase PKR

Kobe C. Yuen, Baoshan Xu, Ian D. Krantz, and Jennifer L. Gerton

Inventory of Supplemental Information

2 supplemental tables

5 supplemental figures

Table S1—Table of primers, related to Figure 1

Table S2--Table of primers, related to Figure 2

Figure S1—related to Figure 1

Figure S2—related to Figure 1

Figure S3—related to Figure 2

Figure S4—related to Figure 4

Figure S5—related to Figure 4

Figure S1

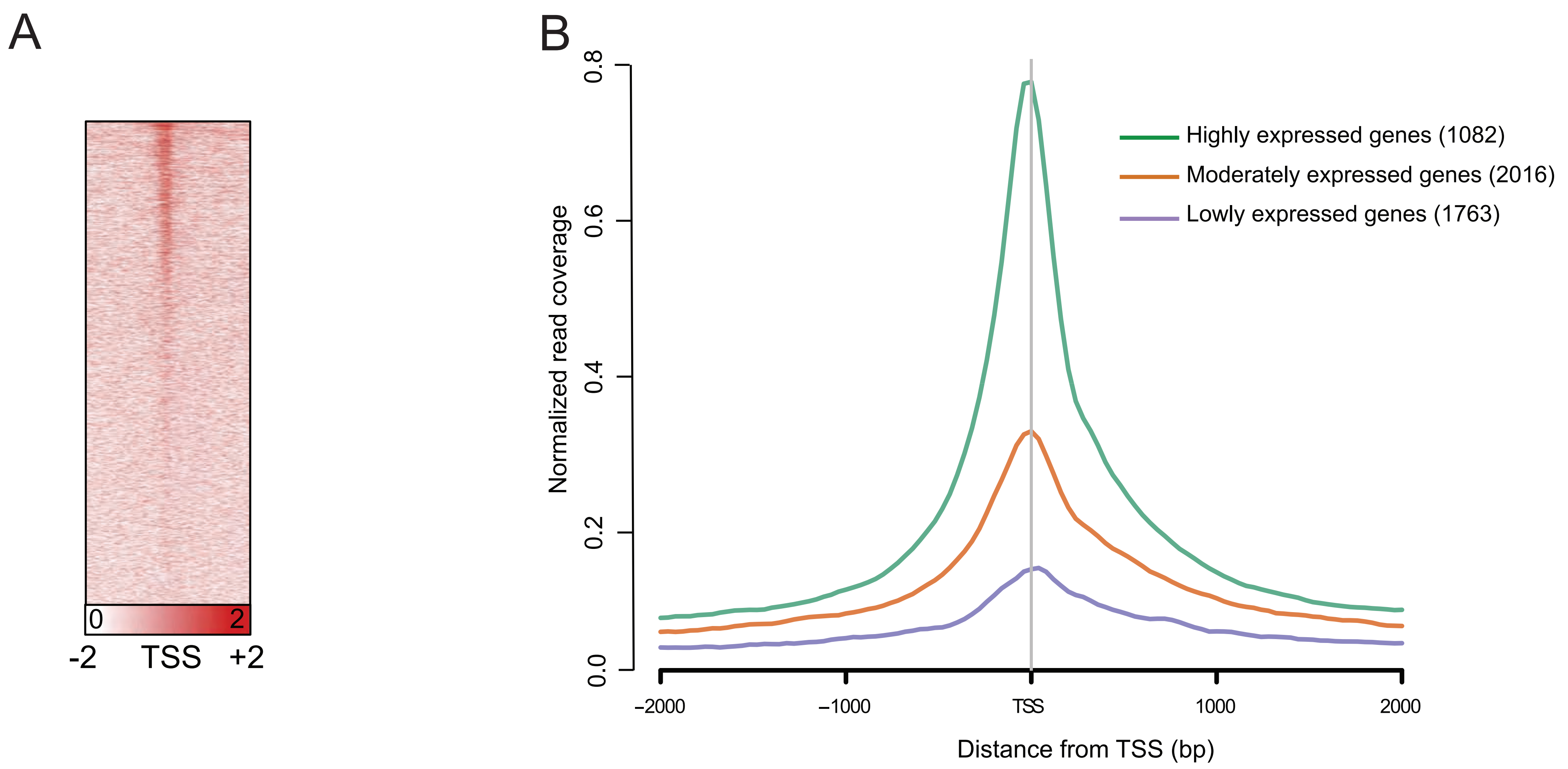
A
0
2
-2
TSS
+2
B
Normalized read coverage
0.0
0.2
0.4
0.6
0.8
−2000
−1000
TSS
1000
2000
Distance from TSS (bp)
Highly expressed genes (1082)
Moderately expressed genes (2016)
Lowly expressed genes (1763)

Figure S2

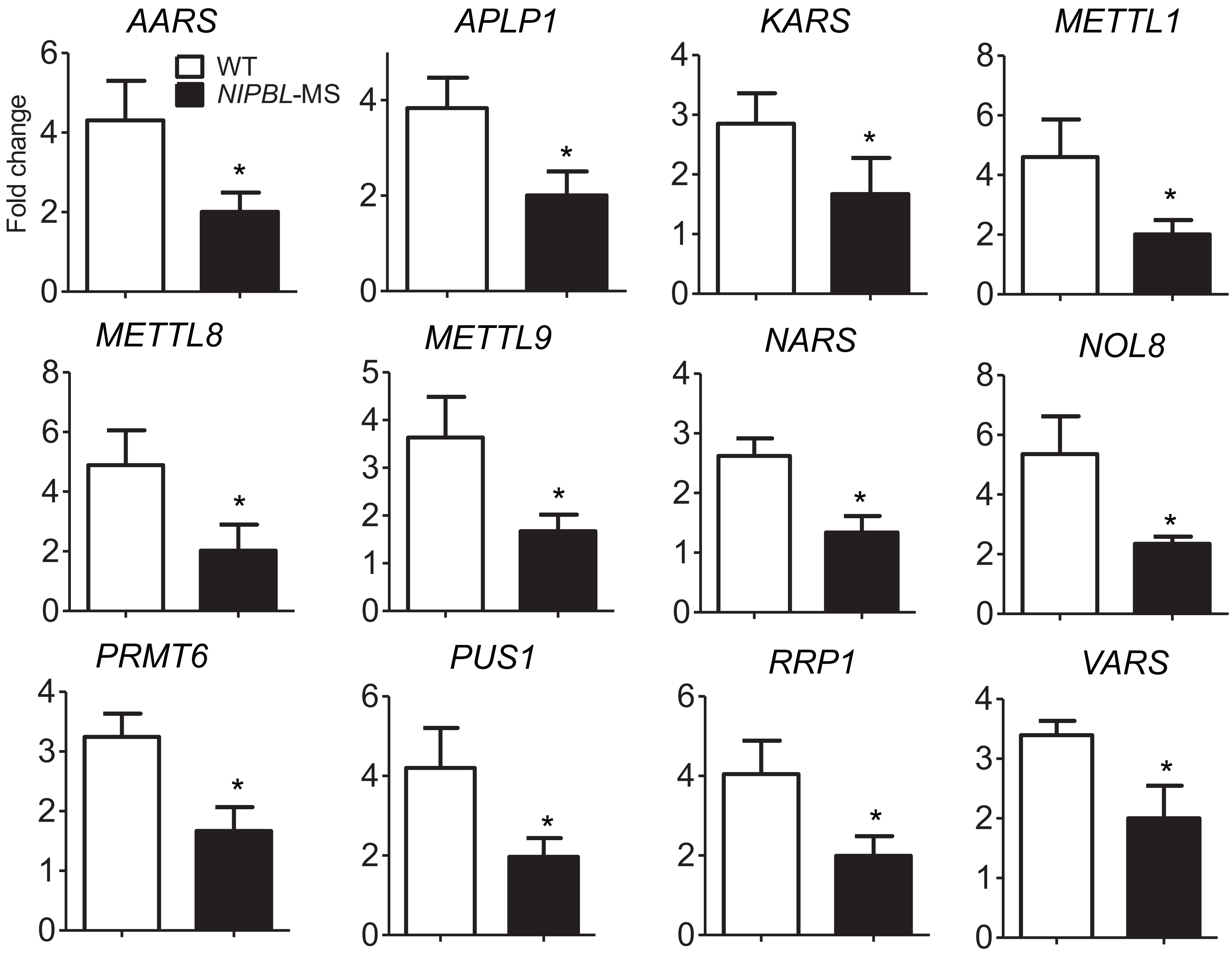

AARS
APLP1
KARS
METTL1
METTL8
METTL9
NARS
NOL8
PRMT6
PUS1
RRP1
VARS
WT
NIPBL-MS
Fold change

Figure S3

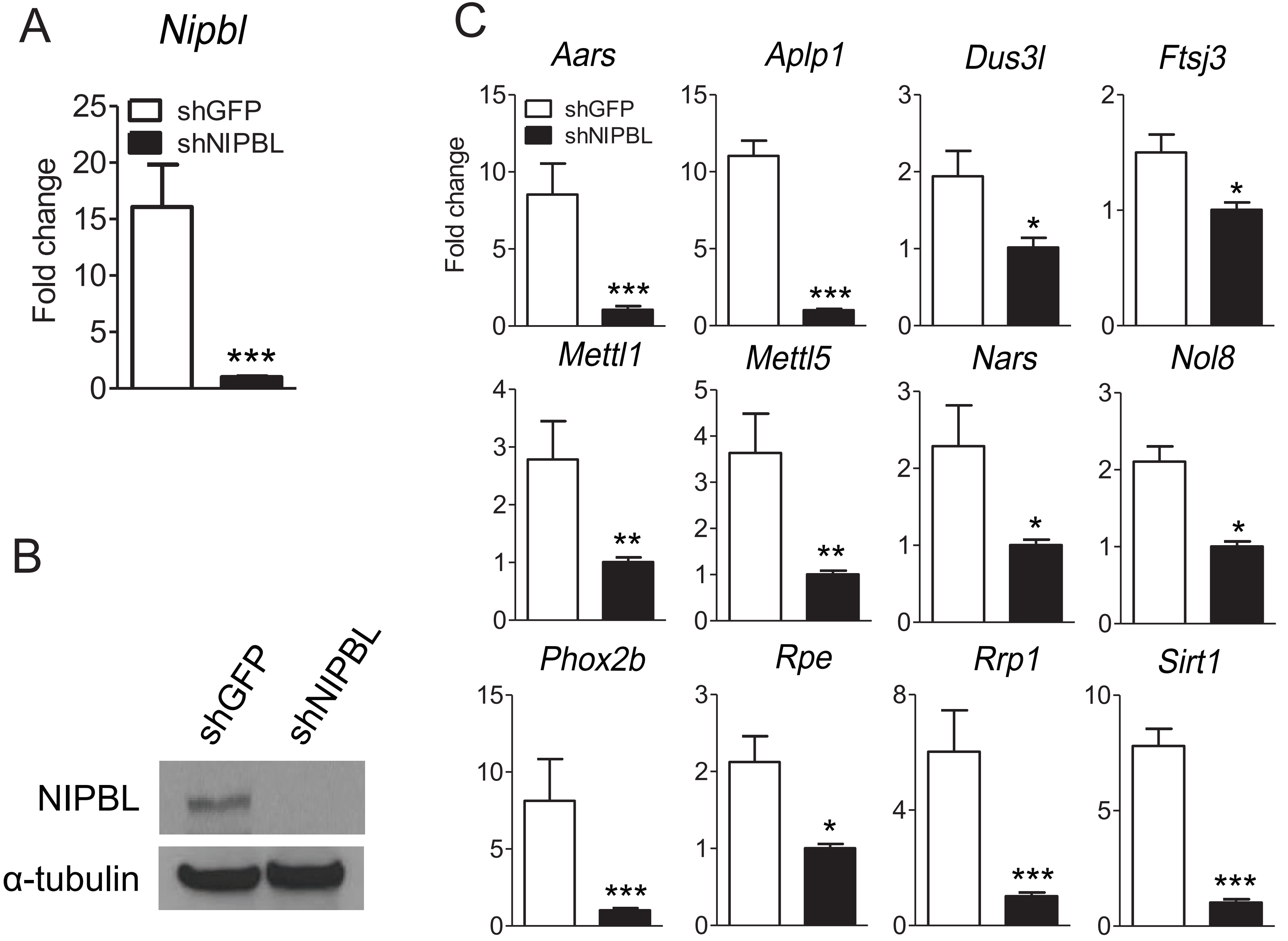
A
Nipbl
shGFP
shNIPBL
Fold change
***
B
shGFP
shNIPBL
NIPBL
α-tubulin
C
Aars
Aplp1
Dus3l
Ftsj3
Mettl1
Mettl5
Nars
Nol8
Phox2b
Rpe
Rrp1
Sirt1
shGFP
shNIPBL
Fold change

Figure S4

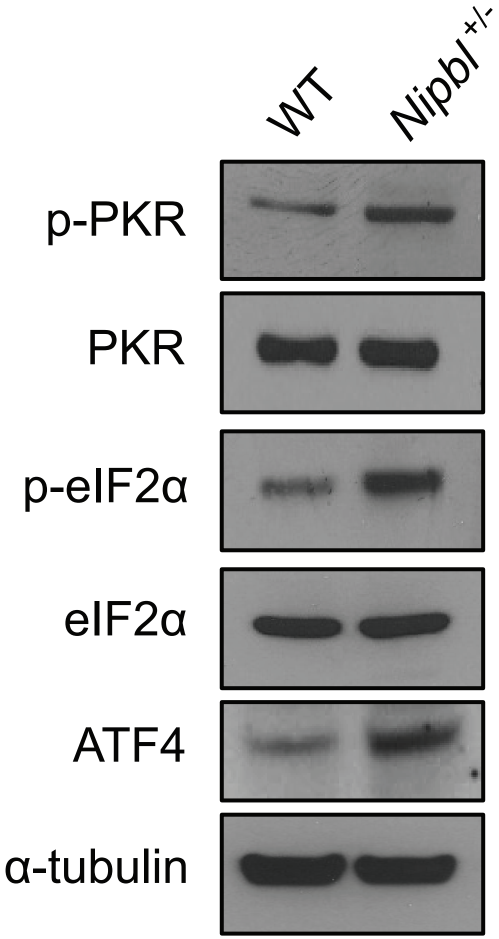

Figure S5

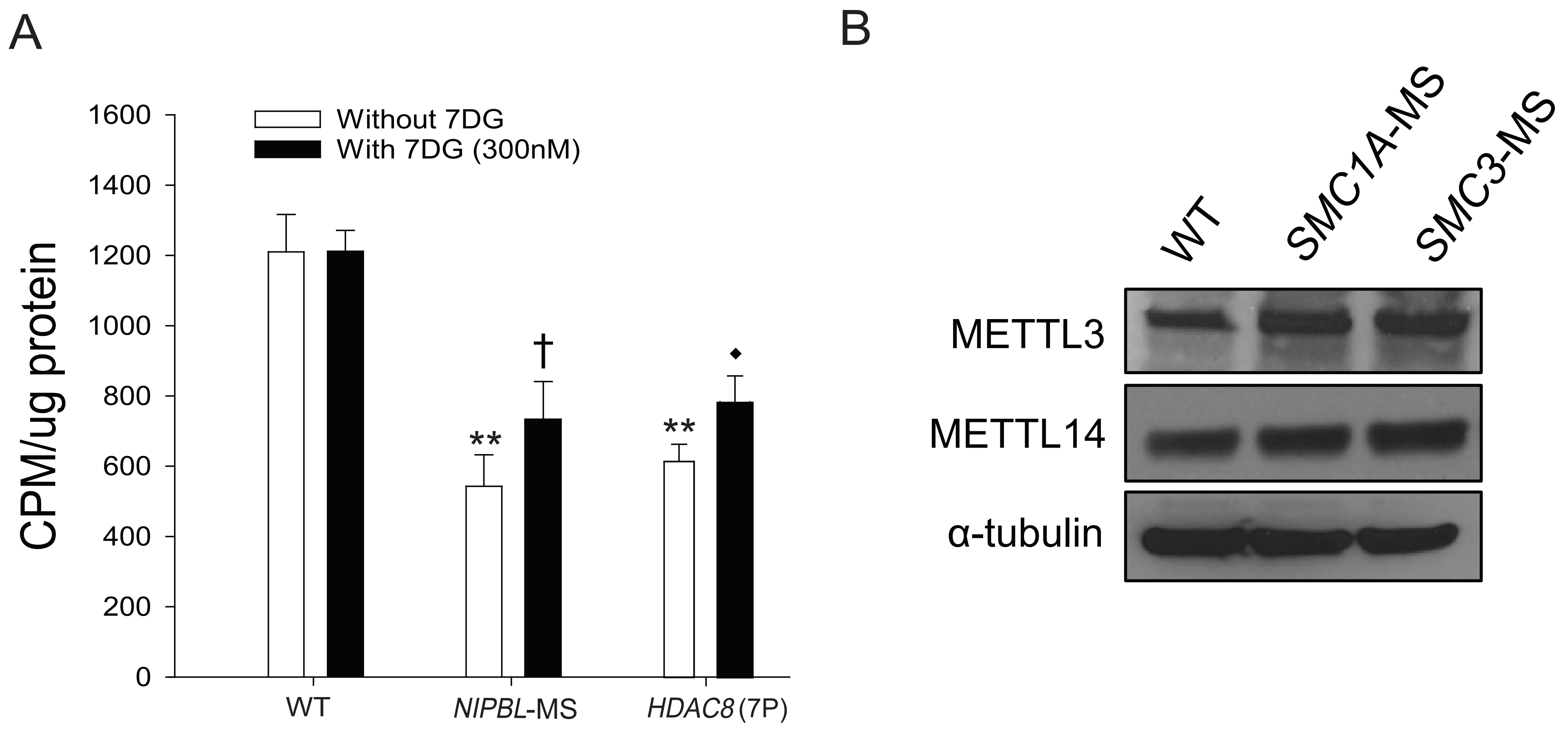

A
1600
1400
1200
1000
800
600
400
200
0
CPM/ug protein
Without 7DG
With 7DG (300nM)
**
†
**
◆
WT
NIPBL-MS
HDAC8 (7P)
B
WT
SMC1A-MS
SMC3-MS
METTL3
METTL14
α-tubulin

Supplemental Figures

Figure S1, related to Figure 1. NIPBL preferentially binds to transcription start site (TSS)/promoter of active genes. (**A**) Heatmap showing alignment of NIPBL peaks at the TSSs from ChIP data collected in LCLs. (**B**) Metagene analysis of NIPBL binding in LCLs according to different levels of gene expression shows that NIPBL mainly binds to TSSs of active genes, with enriched binding at highly expressed genes. The number of genes in each group is shown in parenthesis.

Figure S2, related to Figure 1. RNA processing genes are expressed at lower levels in *NIPBL*-MS LCLs. The lower expression of 12 RNA processing genes identified from the heatmap of Figure 1C was confirmed by qPCR. The gene expression levels were normalized to housekeeping genes, either *ACTB* or *GAPDH*. *P<0.05 compared to WT.

Figure S3, related to Figure 2. The expression of RNA processing genes is significantly decreased upon *Nipbl* shRNA knockdown in mESCs. The reduced expression of *Nipbl* following knockdown is confirmed by both (**A**) qPCR and (**B**) Western blot. (**C**) qPCR shows reduced expression of some of the RNA processing genes in the heatmap from Figure 2C. The gene expression levels were normalized to housekeeping genes, either ATP synthase β-subunit (*Atp5B*) or ubiquitin C (*Ubc*). *P<0.05, **P<0.01 and ***P<0.001 compared to shGFP control.

Figure S4, related to Figure 4. PKR activation in *Nipbl*$^{+/-}$ MEFs. p-PKR, p-eIF2α and ATF4 levels are significantly increased in *Nipbl*$^{+/-}$ MEFs, compared to the levels in MEFs derived from WT littermates, suggesting that the PKR signaling cascade is up-regulated.

Figure S5, related to Figure 4. 7DG partially restores protein synthesis in *NIPBL*-MS and *HDAC8* LCLs. (**A**) Protein synthesis in WT, *NIPBL*-MS and *HDAC8* (7P) LCLs was determined by a ^{35}S methionine metabolic labeling assay. There was decreased protein synthesis in *NIPBL*-MS and *HDAC8* (7P) LCLs, which was significantly improved by treatment with 7DG. **P<0.01 compared to WT, without 7DG; †P<0.05 compared to *NIPBL*-MS, without 7DG; ◆P<0.05 compared to *HDAC8* (7P), without 7DG. (**B**) METTL3 and METTL14 protein expression is not significantly changed in *SMC1A*-MS and *SMC3*-MS LCLs, compared to the WT control.

Table S1, related to Figure 1. Human qPCR Primers

Primer	Sequence (5’-3’)	Amplicon size (bp)
Aars_F	TCCGGCAGCGATTTATAGATTTC	107
Aars_R	GCCTGCATTGGCAAAGAGC	
Aplp1_F	GGACCAATGTGAGAGTTCAACC	105
Aplp1_R	GAGCCACAGGGTAAGAGCA	
Kars_F	GAGCTGAGCATCATTCCGTATG	103
Kars_R	GGCGATACCTTGTTTCCTTGT	
Mettl1_F	CCGACCCACATTTCAAGCG	121
Mettl1_R	TCCAGCACATCGGTTATGGTA	
Mettl3_F	CATTGCCCACTGATGCTGTG	82
Mettl3_R	AGGCTTTCTACCCCATCTTGA	
Mettl8_F	TGAAGAGAAGGCGAGAGAATCA	87
Mettl8_R	GGCACAGTAGGACAGTGCAT	
Mettl9_F	AATTATGCGAATCACTCCAGGC	91
Mettl9_R	CCCGATTTCTCAATGCTGTTGTT	
Mettl14_F	GAACAGAGCTTAAATCCCCA	99
Mettl14_R	TGTCAGCTAAACCTACATCCCTG	
Nars_F	CTGGTGTTGCGAGATGGTACA	88
Nars_R	CCGTGGACAAGAGAACTCCATT	
Nol8_F	TCTGCACAGATTGGCCCAAG	83
Nol8_R	TTTCTAACAAGTTGGCGTTACCT	
Prmt6_F	GGAGTCGGAGAAACCCCTG	118
Prmt6_R	TGAAACGTCCGTGTCTTGCTC	
Pus1_F	GGCCAGGTGGTATCCCTGA	89
Pus1_R	CCCAGAATCCGAATGTGAGAGG	
Rrp1_F	CAGGTGGTTTTACGCACGAC	120
Rrp1_R	GAACGAGCTGGGAAATAGTCC	
Vars_F	AGAAAGGTGACCGGATTTACCA	96
Vars_R	CTGCTGAGAGTTTAGGGTCCA	
Actb_F	ACCTTCTACAATGAGCTGCG	62
Actb_R	CCTGGATAGCAACGTACATGG	
Gapdh_F	ACATCGCTCAGACACCATG	78
Gapdh_R	TGTAGTTGAGGTCAATGAAGGG	

Table S2, related to Figure 2. Mouse qPCR Primers

Primer	Sequence (5'-3')	Amplicon size (bp)
Aars_F	TCCAGTATAACAGGTAGTGTGGG	105
Aars_R	CCAAAAGTAAAGATCGCAGACCA	
Aplp1_F	GCCACTGTCATTGCTGCTTC	122
Aplp1_R	GGGTTAGACGCCCACATAGTC	
Bop1_F	GACTCCAGTCTCTCTGACAGC	122
Bop1_R	CTATGGTTGTCTTCGTCACCG	
Dus3l_F	ATTAAAGCTCAGTACCGCACAA	187
Dus3l_R	CCTGCCTTTCACGGGATTCAA	
Ftsj3_F	CTGCGAAGTCTCTACAAGAAAGC	143
Ftsj3_R	TTCATCCGAGAGTCCACTACC	
Mettl1_F	CAGACCACACACTGCGCTA	114
Mettl1_R	CATCCTTTGGATCATCATGGCTC	
Mettl5_F	AACTAGAGAGTCGCCTGCAAG	147
Mettl5_R	CTGCAACCGCTTTGTTTTCAA	
Nipbl_F	ACCAGCAAGACGATATGCTATCT	114
Nipbl_R	CCTGTGGAGTAATGGGTGTTTTT	
Nars_F	GAGCTGTATGTATCTGACCGAGA	121
Nars_R	AAATGGTGGGAAATGGCTCTTT	
Nol8_F	CCAGCAATCTTGATAGATGCCC	115
Nol8_R	CCGAACTGATTTTGTAGGTCTGT	
Phox2b_F	GGGCTAAGTTTCGCAAGCAG	138
Phox2b_R	CAGTGCTGTCGGGATCAGTG	
Rpe_F	GCACCTGGATGTAATGGACGG	89
Rpe_R	CCTGGCCTAGCTGCTTTCG	
Rrp1_F	GCGGGCAATGAACAGGTGA	62
Rrp1_R	GCCTCGATGTACTTCCGCAG	
Sirt1_F	GCTGACGACTTCGACGACG	101
Sirt1_R	TCGGTCAACAGGAGGTTGTCT	
Atp5b_F	TTGACAACATCTTCCGCTTTACC	66
Atp5b_R	AAGGGATTCTGCCCAATAAGG	
Ubc_F	GCCCAGTGTTACCACCAAGAAG	73
Ubc_R	GCTCTTTTTAGATACTGTGGTGAGGAA	
